# Serum Biomarkers for Early Detection of Gynecologic Cancers

**DOI:** 10.3390/cancers2021312

**Published:** 2010-06-14

**Authors:** Yutaka Ueda, Takayuki Enomoto, Toshihiro Kimura, Takashi Miyatake, Kiyoshi Yoshino, Masami Fujita, Tadashi Kimura

**Affiliations:** Department of Obstetrics and Gynecology, Osaka University Graduate School of Medicine, 2-2, Yamadaoka, Suita, Osaka, 565-0871, Japan; E-Mails: ZVF03563@nifty.com (Y.U.); kimbinbin@gmail.com (T.K.); tmiyatake@aim.com (T.M.); yosino-ki@mc.pref.osaka.jp (K.Y.); fujita@gyne.med.osaka-u.ac.jp (M.F.); tadashi@gyne.med.osaka-u.ac.jp (T.K.)

**Keywords:** ovarian, endometrial, cervical, cancer, serum, tumor marker, CA 125, SCC, proteomics, biomarkers

## Abstract

Ovarian, endometrial, and cervical cancers are three of the most common malignancies of the female reproductive organs. CA 125, historically the most reliable serum marker for ovarian cancer, is elevated in 50% of early-stage ovarian tumors. For endometrial cancers, there are no established serum markers. SCC, which is the best studied serum marker for squamous cell carcinomas, has been unreliable; SCC is elevated in cervical squamous cell carcinomas ranging from 28–85% of the time. Recent proteomics-based analyses show great promise for the discovery of new and more useful biomarkers. In this review, we will discuss the currently utilized serum tumor markers for gynecologic cancers and the novel biomarkers that are now under investigation.

## 1. Introduction

Endometrial, cervical, and ovarian cancers are the three most common malignancies of the female reproductive tract. In the United States alone, roughly 12,000 women are diagnosed with uterine cervical cancer annually, and 4,000 will die from the disease [1]. The relatively low incidence of cervical cancer in the US is largely attributable to the effectiveness of Papanicolaou’s cytological cervical screening test (the Pap test). The importance of screening for early tumor markers is supported by the finding that 60% of cervical cancers occur in women who have never received a Pap test, or who have not been tested in the past five years [1]. For comparison of effectiveness, the serum marker SCC, which is the best studied serum marker for squamous cell carcinomas, is elevated in only 22–60% of early-stage cervical squamous cell carcinomas [2,3]. 

Endometrial cancer is even more common than cervical cancer in the US, with 37,000 diagnosed with it and 7,000 deaths attributable to it in 2005, making it the fourth most common cancer in women [1]. In approximately 75% of endometrial cancer cases, the tumor remains confined to the uterus (FIGO stage I) and has a favorable prognosis. The prognosis, however, worsens dramatically as the disease progresses [4]. Despite this, screening for endometrial cancer is not currently done because of the lack of an appropriate, cost-effective, and acceptable test [5].

Intermediate in frequency between cervical and endometrial cancers, but by far the most fatal, is ovarian cancer. In 2005, 20,000 women in the US were diagnosed with ovarian cancer, but a heartbreaking 15,000 (75%) of these women died from the disease [1]. Roughly three-quarters of ovarian cancer cases present at an advanced disease stage, with the disease spread well beyond the ovaries [6]. In advanced-stage disease, patients most often have first symptoms related to the presence of an enlarging tumor and ascites fluid. However, in early- and mid-stage disease, most patients are asymptomatic for a prolonged period [5]. Serum cancer antigen-125 (CA 125) levels and transvaginal ultrasonography (TV-USG) can contribute to the early detection of ovarian cancer. Unfortunately, these tests are not currently cost-effective; they are thus not used routinely to screen for ovarian cancer [5]. 

For ovarian, endometrial, and cervical cancers, it is critical to detect the disease at the earliest possible stage. The discovery of useful serum biomarkers for the early detection of gynecologic cancers has thus been a high priority. Such tumor markers will be molecules arising from the presence of a tumor, which can appear in the surrounding tissue, and then within the blood and excretions. 

Tumor markers can be secreted or shed by the tumor in excess of the normal tissue or cell phenotype. Sometimes, the molecule is uniquely specific to the tumor phenotype, often as embryonic, fetal (*i.e*., AFP), undifferentiated, or stem-cell phenotypes. These can occur as re-expression of silenced genes or as an alternative mRNA splicing expression of an already expressed gene product. Some glycoproteins produced by cancer cells have altered glycan structures, although the proteins themselves are ubiquitous [7]. Tumor markers might be unique extracellular matrix or cell adhesion molecules, receptors, growth factors, cytokines, or products of abnormal metabolism. Rarely, the marker molecules can be released by other tissues and organs in response to signals from the tumor. Even the body’s own antibodies against tumor markers can be markers. 

Tumor markers are secreted, released, or leaked into the interstitial fluids, and thus into the lymph, and finally (or directly) into the bloodstream, where they become detectable in serum samples. To be able to enter the bloodstream directly, larger molecules, often proteins, are cleaved into truncated forms or fragments, which are sometimes specific to the protease micro-environment of the tumor.

Tumor markers can be associated with patient diagnosis, prognosis, clinical management, and follow-up. Ideally, a serum marker would only appear in the blood of patients with a true malignancy; the marker would correlate with tumor stage and response to treatment, and it could be easily, cheaply, and reproducibly measured. The serum marker would be used for the screening of healthy populations and of specific groups with higher risk factors. The marker would enable a diagnosis for a specific type of cancer, help determine prognostic factors, and be used to monitor the course of treatment, remission, and recurrence, while receiving surgery, radiation, chemical, and immunological treatments. 

Recent advances in clinical proteomics have propelled us into an exciting period of discovery of new cancer biomarkers, although the available proteomic technologies have their limitations. The principles of proteomic technology require stringent guidelines for the collection of clinical material, the application of analytical techniques, and for our interpretation of the data.

In this review, we present an overview of the serum tumor markers in current use. A lack of sensitivity and specificity has, so far, given most of the tumor markers in current use an unsatisfactory predictive value. We will discuss the novel biomarkers of the future, where there is great hope for the better detection and management of gynecologic cancers, including ovarian, endometrial, and cervical cancers.

## 2. Serum Markers for Cervical Cancer

Screening for cervical cancer with cervical cytology reduced the incidence of cervical cancer by more than 50% over the past 30 years in the United States [8]. However, it is estimated that 50% of the women in whom cervical cancer is diagnosed each year will have never had cervical cytology testing [8]. One approach for further reducing the incidence and the mortality of cervical cancer would be to increase the screening rates among groups of women at highest risk, who currently are not being screened. Another would be the establishment of appropriate serum testing for the early detection of cervical cancer. The squamous cell carcinoma antigen, SCC, is the most commonly used serum marker for squamous cell cervical carcinoma, which makes up 85–90% of all cervical carcinomas [4]. Elevated serum SCC levels have been detected in 28–88% of cervical squamous cell carcinomas [9,10,11,12,13,14,15] (Table 1). Pre-treatment levels of SCC have been shown to be related to the stage of the disease, size of the tumor, depth of the stromal invasion, the lymph-vascular space involvement, and lymph node metastasis [9,11,13,14,16,17,18,19]. Elevated SCC levels were also demonstrated to have predictive value for prognosis in some studies [16,17,19]. 

**Table 1:** Diagnostic serum markers for cervical cancer in clinical use.

Serum markers	SCC	CYFRA 21-1	CA 125	CA 19-9	CEA	IAP
Positive rates	squamous	squamous	adeno	adeno	adeno	43–51%
	28–85%	42–52%	27–75%	35–42%	26–48%	

Positive rates (elevated serum levels) detected for the indicated serum markers, in cases of squamous cell carcinoma (squamous), adenocarcinoma (adeno), or for all histological types.

Serum SCC levels have also been shown to parallel the responses to radiotherapy and chemotherapy [13,19,20,21]. Elevated pre-radiotherapy SCC levels were detected in 60% of 72 squamous cell carcinoma patients, whereas post-treatment SCC levels were above the cut-off value in only 2% of the patients whose disease was considered completely treated [13]. The serum SCC level also proved to be an independent predictor of response to neoadjuvant chemotherapy in locally advanced cervical cancer patients who received neoadjuvant chemotherapy and radical surgery [19]. SCC has also been used in the follow-up examination of cervical cancer patients. Increased serum SCC was shown to precede the clinical detection of recurrence of the disease [12,13,22].

The marker CYFRA 21-1 (serum fragments of cytokeratin 19) is also being used as a serum tumor marker for cervical cancer, especially for squamous cell carcinoma. Elevated CYFRA 21-1 levels have been detected in 42–52% of patients with squamous cell carcinoma of the uterine cervix [18,23,24] (Table 1). Similar to the usefulness of SCC, pre-treatment levels of CYFRA 21-1 are related to stage of the disease, size of the tumor, depth of the stromal invasion, the lymph-vascular space involvement, and lymph node metastasis [25,26,27]. However, raised CYFRA 21-1 levels were not demonstrated to be of predictive value for prognosis in some studies [18]. 

Serum CYFRA 21-1 levels were reported to be useful for monitoring the response to radiotherapy and chemotherapy [12,24]. The CYFRA 21-1 assay was also used in the follow-up examination of cervical cancer patients. Additionally, increased serum CYFRA 21-1 was shown to precede the clinical detection of recurrence of the disease [12]. 

Adenocarcinoma accounts for 10–15% of cervical cancers [4]. SCC, CYFRA 21-1, CA 125, CA 19-9 and CEA are positive in 20–25%, 33–63%, 27–75%, 34–42% and 26–48%, respectively, of such tumors [9,10,11,20,23,28,29,30,31,32,33,34] (Table 1). Raised serum CA 125 is associated with the stage of the cervical disease and is of some prognostic significance [29]. 

Another novel marker (that is also a target for new drug development) is immunosuppressive acidic protein (IAP), which is elevated in 43–51% of cervical carcinomas [33,34] (Table 1). Battaglia *et al.* found pre-treatment-elevation of serum IAP in 53% of squamous cell carcinoma cases, and in 40% of adenocarcinoma patients [34]. IAP level is related to disease stage and lymph node metastasis, and is of predictive value for prognosis [34]. 

There are some newer serum markers still under investigation (Table 2). Suzuki *et al.* showed that serum M-SCF was elevated in 27% of cervical cancer cases [35]. YKL-40, also known as CHI3L1 or human cartilage glycoprotein-39, was shown to be a potential biomarker in the detection and management of cervical cancer [28]. Elevated serum YKL-40 was found in 75% of squamous cell carcinoma patients and 78% of adenocarcinoma patients. Moreover, elevated pre-treatment-levels of YKL-40 were shown to predict an unfavorable prognosis, independent of the stage of the disease. Significantly elevated serum levels of circulating soluble Fas (sFas) were demonstrated in squamous cell carcinomas when compared to that of healthy women (p < 0.0001) [36]. Vascular endothelial growth factor (VEGF), especially the VEGF-C isoform, was revealed to be elevated in the serum of patients with squamous cell carcinoma and cervical intraepithelial neoplasia (CIN) when compared to that of healthy women [37,38]. In cervical squamous cell carcinoma patients, serum VEGF levels were associated with the stage of the disease, but not with prognosis [38]. The serum level of thymidine kinase (TK) was demonstrated to be significantly higher in patients with cervical carcinoma than in normal women and patients with carcinoma in situ (p < 0.01 and p < 0.05, respectively) [39]. 

**Table 2:** Diagnostic serum markers for cervical cancer currently under investigation.

Serum markers	M-CSF	YKL-40	sFas	VEGF	TK
Positive rates	27%	squamous adeno	squamous	squamos	N/A
		75% 78%	N/A	N/A	

Positive rates (elevated serum levels) detected for the indicated serum markers, in cases of squamous cell carcinoma (squamous), adenocarcinoma (adeno), or for all histological types.

## 3. Serum Markers for Endometrial Cancer

In current practice, screening for endometrial cancer is not undertaken because of the lack of an appropriate, cost-effective, and acceptable test that actually reduces mortality [5]. Routine use of an endometrial cytological test, comparable to the Pap test for cervical cancer, is too insensitive and nonspecific to be useful in screening for endometrial cancer [5]. Currently used serum markers and novel biomarkers under investigation for endometrial cancer are discussed below.

Elevated serum CA 125 levels have been detected in 11–43% of endometrial cancers [40,41,42,43,44,45,46] (Table 3). Pre-treatment CA 125 levels were shown to be related to the stage of the disease, the depth of myometrial invasion, peritoneal cytology, and lymph node metastasis [42,43,44,45,46,47,48]. Raised CA 125 levels were also demonstrated to be of predictive value for prognosis in some studies [42,47]. The serum CA 125 level usually parallels the clinical course of the disease [43,45,49]; however, the fact that serum CA 125 levels are often elevated in disease-free endometrial cancer patients who have undergone abdominal radiation should be kept in mind [50].

**Table 3:** Diagnostic serum markers for endometrial cancer in clinical use.

Serum markers	CA 125	CA 19-9	CA 15-3	CA 72-4	CEA	IAP
Positive rates	11–43%	22–24%	24–32%	22–32%	14–22%	55–76%

Positive rates (elevated serum levels) detected for the indicated serum marker in cases of endometrial cancer are shown.

The serum markers CA 19-9, CA 15-3, CA 72-4, and CEA levels are raised in endometrial cancer patients in 22–24%, 24–32%, 22–32% and 14–22% of the cases, respectively [11,33,40,42,45,51,52] (Table 3). Serum CA 15-3 levels were shown to be associated with prognosis [42,53]. Elevated serum IAP levels have been detected in 55–76% of endometrial cancer patients [17,33] (Table 3). 

There are other serum markers for endometrial cancer now under investigation (Table 4). Raised serum M-SCF levels were detected in 25–73% of endometrial cancer cases [35,43]. HE4 was demonstrated to provide 46% sensitivity for endometrioid adenocarcinoma of the endometrium in all stages at 95% specificity [54]. For stage I disease, HE4 yielded a 17% improvement in sensitivity compared with that of CA 125. Significantly elevated levels of serum sFas were demonstrated in endometrioid adenocarcinoma of the endometrium compared to that of healthy women (p < 0.0001) [36]. Human serum amyloid A (SAA) is a high-density lipoprotein that has recently been proposed as a useful biomarker for several kinds of tumors, including gastric, lung, pancreatic, and nasopharyngeal cancers [55,56,57,58,59]. Cocco *et al.* showed that SAA was overexpressed and actively secreted by Grade-3 endometrioid adenocarcinoma and serous papillary carcinoma of the endometrium, and that SAA was present at high concentration in the serum of these patients [55,60]. 

**Table 4:** Diagnostic serum markers for endometrial cancer under investigation.

Serum markers	M-CSF	HE4	sFas	SAA
Positive rates	25–73%	E	E	G3-E/S
		46%	N/A	N/A

Positive rates (elevated serum levels) of each serum marker detected in cases of squamous cell carcinoma, adenocarcinoma, and for all histological types, are shown. E: endometrioid adenocarcinoma; G3-E: endometrioid adenocarcinoma Grade-3; S: serous adenocarcinoma; N/A: not assessed

Recently, panels of novel biomarkers have been developed to better detect cancers, including endometrial tumors (Table 5). Farias-Eisner *et al.* constructed a multiple logistic regression model (MLRM) with the use of the values for ApoA-1 (apolipoprotein A-1), TF (transferrin), and TTR (transthyretin) for the detection of endometrial cancer [61]. This panel distinguished normal samples from early-stage endometrial cancer with a sensitivity of 71% and a specificity of 88% Additionally, the panel distinguished normal samples from late stage endometrial cancer with a sensitivity of 82% and a specificity of 86%. 

**Table 5:** Novel biomarker panels for detection of endometrial cancer.

Reference		Biomarkers		Sensitivity		Specificity
Zhu *et al.* (2006)[63]		13 proteins		93%		100%
Farias-Eisner *et al.* (2009) [61]		3 proteins		Early Late		Early Late
				71% 82%		88% 86%
Takano *et al.* (2009) [62]		2 proteins		82%		86%

Shown here is the sensitivity and specificity of each serum biomarker panel used in the detection of endometrial cancer; Early: early-stage cases; Late: late-stage cases

Recent proteomic techniques, which can identify differentially expressed proteins in a large set of samples, have been applied to the discovery of new biomarkers in many diseases [62]. Zhu *et al.* has established a diagnostic system with 13 novel potential biomarkers, using surface-enhanced laser desorption-ionization time-of-flight mass spectrometry (SELDI-TOF-MS) to differentiate endometrial cancer patients from healthy women. The technique had a sensitivity of 93% and a specificity of 100% [63]. Takano *et al.* also used SELDI-TOF-MS to identify candidate markers for endometrial cancer [62]. Two of the candidates turned out to be apolipoprotein A-1 and apolipoprotein C-1. Dual-biomarker analysis for the detection of endometrial cancer yielded a sensitivity of 82% and a specificity of 86%. These studies, which analyzed test samples consisting of a significantly higher proportion of cancer patients than would be found in a general population, and thus a significantly lower proportion of healthy controls, had methodological limitations, as previously described [64]. The prevalence of endometrial cancer was 50% in the study by Takano *et al.* [62] and 57% in the study by Zhu *et al.* [63]. Thus, these results might have overstated the sensitivity of the tests. However, because one of the candidate proteins discovered in a study by Takano *et al.* [62], apolipoprotein A-1, was also a constituent of the biomarker panel constructed by Farias-Eisner *et al.* [61], this protein may be a very promising biomarker for endometrial cancer. 

## 4. Serum Markers for Ovarian Cancer

Establishment of an appropriate screening test for ovarian cancer has long been sought. This disease is the leading cause of death from gynecologic malignancies in the US, with its poor prognosis resulting from the lack of reliable, sensitive screening tests and our limited understanding of the mechanisms of chemoresistance and relapse. More than two-thirds of cases of ovarian cancers are diagnosed only after the disease has progressed to stage III or IV and involve the peritoneal cavity or other organs. 

Symptoms that are associated with ovarian cancer are typically nonspecific and the association is often not recognized until the disease is advanced [64]. Previous studies showed that ultrasonography (USG), with or without power Doppler, provided high sensitivity. However, ultrasonography’s specificity and positive predictive values were unsatisfactory [65,66]. Serum markers and novel biomarkers for early detection of ovarian cancer that are currently used or under investigation are discussed below. 

Elevated serum CA 125 levels have been detected in 50% and 92% of ovarian cancers in early and late stages, respectively [67] (Table 6). According to a review by Nossov *et al.*, the positive predictive value of the CA 125 assay for the early detection of ovarian cancer is 57% [68]. Elevated CA 125 occurs in other cancers, such as in endometrial, breast, pancreatic, gastrointestinal, and lung cancers. Raised CA 125 levels are sometimes found in patients with benign gynecologic conditions, such as menstruation, pregnancy, endometriosis, and pelvic inflammatory disease, and even in non-gynecologic conditions, such as hepatitis and pancreatitis [49]. The predictive value of pre-treatment CA 125 levels for prognosis is controversial; however, changes in CA 125 levels correlate with the regression, stability, and progression of the disease in 87–94% of instances [49]. 

**Table 6:** Diagnostic serum markers for ovarian cancer in clinical use.

Serum markers	CA 125	CA 19-9	CA 15-3	CA 72-4	IAP
Positive rates	Early Late	M non-M	50–56%	63–71%	70–93%
	50% 92%	68–83% 28–29%			

Positive rates detected for each serum marker in cases of ovarian cancer are shown. Early: early-stage cases; Late: late-stage cases; M: mucinous adenocarcinoma; Non-M: histological types of ovarian carcinoma other than mucinous adenocarcinoma.

Serum levels of CA 19-9 (a monosialoganglioside antigen widely used in GI adenocarcinoma diagnostics) are elevated in 68–83% of mucinous ovarian cancers, and only in 28–29% of non-mucinous types. In contrast, whereas CA 125 is elevated in 80% of non-mucinous ovarian tumors [69,70,71,72]. Serum CA 15-3, CA 72-4, and CEA levels are raised in 50–56%, 24–32%, 63–71% and 70–93% of the ovarian cancer patients, respectively [71,73,74,75,76,77,78] (Table 6). According to Gadducci *et al.*, CA 19-9, CA 15-3, and CA 72-4 correlated worse than CA 125 with the clinical course of the disease. Additionally, these markers did not offer additional clinical benefit for monitoring ovarian cancer, suggesting that the serial measurement of these markers may play a role only in the management of patients with a normal CA 125 assay [49]. 

There are serum markers for ovarian cancer that are under active investigation (Table 7). In a review by Li *et al.*, HE4 displayed the highest sensitivity among single markers, including CA 125, in the detection of ovarian cancer in both the early (62–83%) and late (75–93%) stages, respectively [79]. Elevated serum lysophosphatidic acid (LPA) levels were found in 90% and 98% of ovarian cancer in early and late stages, respectively. However, serum levels of LPA do not correlate with the stage of the disease, and nonspecific elevation of LPA was detected in healthy and benign gynecologic conditions [68,80,81]. Significantly elevated sFas levels were detected in ovarian cancer patients when compared to that of healthy women. Additionally, the serum sFas level was demonstrated to be a statistically significant indication factor for survival, as well as for the histological grading of ovarian carcinomas [36]. 

**Table 7:** Diagnostic serum markers for ovarian cancer under investigation.

Serum markers	HE4	LPA	sFas
Positive rates	Early Late	Early Late	N/A
	62–83% 75–93%	90% 98%	

Positive rates detected for each serum marker in cases of ovarian cancer are shown. Early: early-stage cases; Late: late-stage cases; N/A: not assessed

Novel biomarker panels have also been investigated for the early detection of ovarian cancers (Table 8). Zhang *et al.* identified a panel that consisted of three proteins, including ApoA-1, a truncated form of TTR, and a cleavage fragment of H4 (inter-α-trypsin inhibitor heavy chain), to detect early-stage ovarian cancer with a sensitivity of 83% and a specificity of 94% [82]. 

**Table 8:** Novel biomarker panels for the detection of ovarian cancer.

Reference		Biomarkers		Sensitivity		Specificity
Zhang *et al.* (2004) [82]		3 proteins		early		early
				83%		94%
Su *et al.* (2007) [83]		4 proteins		early, M early, all		early, M early, all
				95% 89%		92% 97%
Nosov *et al.* (2009) [84]		4 proteins		early, S early, E		early, S early, E
				94% 98%		94% 98%
Visintin *et al.* (2008) [85]		6 proteins		95%		99%

The sensitivity and specificity of each serum biomarker panel used for the detection of ovarian cancer are shown; Early: early-staged cases; Late: late-staged cases; All: cases in all stages; S: serous adenocarcinoma; M: mucinous adenocarcinoma.

Su *et al.* utilized an MLRM with values for CA 125, ApoA-I, TF, and TTR for the early detection of ovarian cancer [83]. This model provided a sensitivity of 89% and a specificity of 97% for the detection of early stage ovarian cancer. Furthermore, the sensitivity and the specificity of this panel in distinguishing between normal and mucinous ovarian cancer samples were 95% and 92%, respectively. Nosov *et al.* applied this same MLRM model and marker panel to serous and endometrioid histological types of ovarian carcinomas and demonstrated a sensitivity of 94% and a specificity of 94% for serous ovarian carcinoma in its early stage, and a sensitivity of 98% and a specificity of 98% for endometrioid ovarian carcinoma in its early stage [84]. 

Visintin *et al.* proposed a set of serum biomarkers that consisted of leptin, prolactin, osteopontin, insulin-like growth factor II (IGFII), macrophage inhibitory factor (MIF), and CA 125 to discriminate between ovarian cancer patients and healthy women. The panel had a sensitivity of 95% and a specificity of 99% [85]. Not surprisingly, this panel provided a significant improvement over CA 125 alone. However, these studies had the same methodological limitations, of excessive numbers of tumor cases versus matched population controls, described above in the endometrial cancer section. With that being said, novel proteomics-based investigations and bioinformatics analyses still provide the greatest promise of using existing approaches to find ever more accurate and useable biomarkers for gynecological cancers. 

One important question remains: how long before the diagnosis of an ovarian cancer does the serum level of various markers begin to rise above background levels as a tumor grows? Anderson *et al.* [86] studied concentrations of CA125, HE4, and mesothelin in serum samples collected from 0–18 years before the diagnosis of a tumor, as part of an unrelated study. They found that the markers may provide some evidence of ovarian cancer up to 3 years before clinical diagnosis, but the more likely lead time for the detection of a change associated with these markers appears to be less than 1 year.

## 5. Conclusions

For gynecologic cervical, endometrial, and ovarian cancers, only a small handful of tumor-associated antigens, such as SCC and CA 125, have been routinely used as tumor markers. Some markers are useful, not only as diagnostic tools, but also as a predictive marker for the prognosis and the clinical course after treatment. Some recently investigated new serum markers seem to be clinically useful, such as YKL-40 for cervical cancer and HE4 for endometrial and ovarian cancers. Recent breakthroughs in proteomics and bioinformatics technology will expand our understanding of tumor-specific biomarkers. Such investigations will establish newer and more useful biomarkers for the more accurate detection and management of ovarian, endometrial, and cervical cancers.

## Abbreviations

ApoA-1: apolipoprotein A-1
CA 15-3: cancer antigen-15-3
CA 19-9: cancer antigen-19-9
CA 72-4: cancer antigen-72-4
CA 125: cancer antigen-125
CEA: cancer embryonic antigen
CIN: cervical intraepithelial neoplasia
CYFRA 21-1: cytokeratin 19 fragments
HE4: (WFDC2), human epididymis-specific 4-disulfide core protein
H4: inter-α-trypsin inhibitor heavy chain fragment
IAP: immunosuppressive acidic protein
IGFII: insulin-like growth factor II
MIF: macrophage inhibitory factor
LPA: lysophosphatidic acid
MLRM: multiple logistic regression model
M-CSF: macrophage colony-stimulating factor
Pap test: Papanicolaou’s test
SAA: human serum amyloid A
SCC: squamous cell carcinoma antigen
SELDI-TOF-MS: surface-enhanced laser desorption-ionization time-of-flight mass spectrometry
sFas: serum soluble Fas
TF: transferrin
TK: thymidine kinase
TTR: transthyretin
TV-USG: transvaginal ultrasonography
USG: ultrasonography
VEGF-C: serum isoform of vascular endothelial growth factor
YKL-40: (aka CHI3L1) human cartilage glycoprotein-39
